# A Dual-Modal Adaptive Pyramid Transformer Algorithm for UAV Cross-Modal Object Detection

**DOI:** 10.3390/s25247541

**Published:** 2025-12-11

**Authors:** Qiqin Li, Ming Yang, Xiaoqiang Zhang, Nannan Wang, Xiaoguang Tu, Xijun Liu, Xinyu Zhu

**Affiliations:** 1 College of Aviation Electronics and Electrical, Civil Aviation Flight University of China, Guanghan 618307, China; fredzhe4455@163.com (Q.L.); xqzhanguestc@163.com (X.Z.); txg198955@163.com (X.T.); liuxijun@cafuc.edu.cn (X.L.); cafuczxy@dingtalk.com (X.Z.); 2 School of Optoelectronic Science and Engineering, University of Electronic Science and Technology of China, Chengdu 610054, China; 3 Sichuan Province Engineering Technology Research Center of General Aircraft Maintenance, Civil Aviation Flight University of China, Guanghan 618307, China; 4 Luoyang College, Civil Aviation Flight University of China, Luoyang 471000, China; 15896555218@163.com

**Keywords:** unmanned aerial vehicle, target detection, dual-modality fusion, transformer, infrared-visible image detection

## Abstract

**Highlights:**

**What are the main findings?**

- A DAP-enhanced YOLOv8 model is proposed to address the bottlenecks of UAV infrared–visible object detection.
- The DAP module effectively improves multi-scale feature representation, low-light robustness, and cross-modal fusion efficiency.

**What are the implications of the main findings?**

- The proposed approach achieves superior detection accuracy and real-time performance on DroneVehicle and LLVIP datasets.
- The method provides a practical solution for UAV-based infrared–visible perception in complex and low-illumination environments.

**Abstract:**

Unmanned Aerial Vehicles (UAVs) play vital roles in traffic surveillance, disaster management, and border security, highlighting the importance of reliable infrared–visible image detection under complex illumination conditions. However, UAV-based infrared–visible detection still faces challenges in multi-scale target recognition, robustness to lighting variations, and efficient cross-modal information utilization. To address these issues, this study proposes a lightweight Dual-modality Adaptive Pyramid Transformer (DAP) module integrated into the YOLOv8 framework. The DAP module employs a hierarchical self-attention mechanism and a residual fusion structure to achieve adaptive multi-scale representation and cross-modal semantic alignment while preserving modality-specific features. This design enables effective feature fusion with reduced computational cost, enhancing detection accuracy in complex environments. Experiments on the DroneVehicle and LLVIP datasets demonstrate that the proposed DAP-based YOLOv8 achieves mAP_50:95_ scores of 61.2% and 62.1%, respectively, outperforming conventional methods. The results validate the capability of the DAP module to optimize cross-modal feature interaction and improve UAV real-time infrared–visible target detection, offering a practical and efficient solution for UAV applications such as traffic monitoring and disaster response.

## 1. Introduction

Unmanned Aerial Vehicles (UAVs), with their flexible aerial perspective and real-time data acquisition capabilities, have been deeply integrated into key application fields such as intelligent traffic flow management [1,2], earthquake disaster emergency response [3], and three-dimensional border security patrols [4]. The pressing need for real-time target localization and semantic comprehension in dynamic monitoring contexts has propelled the inventive use of computer vision technology in UAV payload systems. Computer vision achieves digital acquisition, feature extraction, and semantic interpretation of scene information via intelligent perception devices. Object identification, as a fundamental task, has emerged as a technical barrier and a focal point of research in UAV intelligent monitoring systems due to its requirement to concurrently address target spatial coordinate regression and category semantic classification [5,6].

Practical applications of UAV payload object detection confront three technical constraints, and present methods still fail to meet them. Initially, multi-scale target representation is inadequate due to varying pixel sizes (10 × 10 to 200 × 200) in monitoring scenarios. Traditional algorithms process micro-targets (with edge lengths < 200 pixels), resulting in background noise obscuring targets. This immediately reduces high-level semantic information in the feature pyramid, and such algorithms’ feature extraction integrity for micro-targets is about 40% poorer than for medium and large targets, which is one of the main causes of missed detections [7]. Second, multi-modality robustness is limited in complex environments, such as night scenes with low RGB values and low texture feature entropy, reducing detection accuracy by 50–70% [8]. In rainy and foggy conditions, infrared thermal imaging heat source positioning errors reach the pixel level, causing over 35% confusion between targets [9]. Third, dual-modality fusion technology has yet to overcome several key bottlenecks. While the architectural evolution from two-stage detectors like Fast R-CNN [10] and Faster R-CNN [11] to single-stage approaches such as YOLO [12] and SSD [13] has significantly improved speed, the inherent flaws of single-modality inputs persist. For instance, early pixel-level fusion is prone to introducing artifacts and noise, particularly from sensor calibration errors, which can substantially increase the false detection rate compared to single-modality methods [14]. Furthermore, mid-stage fusion based on CNNs struggles to effectively capture long-range cross-modal spatial correlations—such as between infrared heat sources and visible light textures—due to the inherently local receptive field of convolution operations. A fundamental challenge here is to leverage the complementary strengths of each modality without introducing noise or feature contamination from the other, a problem that naive fusion struggles with. Consequently, the rate of missed detections in complex environments like urban blocks remains stubbornly high and has not seen significant reduction [15,16]. These persistent challenges in cross-modal complementarity, multi-scale aggregation, and adaptability highlight the urgent need for new technical approaches in feature fusion and representation.

To address these challenges, we propose a novel framework guided by a two-stage fusion philosophy. First, ensure strict modal independence during initial feature extraction to preserve the integrity of modality-specific patterns; second, introduce a joint attention mechanism that acts as an adaptive gating system, selectively integrating features based on their contextual relevance rather than blindly mixing them. This ensures robust fusion by dynamically suppressing irrelevant or noisy information from either modality. Our primary contributions are threefold.

Firstly, we propose a Dual-modality Adaptive Pyramid Transformer (DAP) module that innovatively integrates 8 × 8 anchor pooling with self-attention mechanisms. This module serves as the core of our adaptive fusion strategy, achieving dual optimization: enhancing tiny target details in the P3 layer through adaptive feature refinement and capturing long-distance contextual dependencies in the P5 layer, while maintaining computational efficiency during cross-modal correlation modeling.

Secondly, we develop a modality-specific dual-stream backbone network based on a variant of CSPDarknet (Cross Stage Partial Darknet). CSPDarknet, the foundational backbone of the YOLO series, is renowned for its efficiency. It partitions the feature map into two parts, with one part undergoing a series of bottleneck transformations while the other is directly concatenated, thereby enhancing the network’s learning capability while significantly reducing computational complexity and memory overhead. This design materializes the principle of modal independence, employing a unified down-sampling pathway with uniform strides and integrating the C2f module to strengthen visible light texture feature extraction, establishing a precise and uncontaminated foundation for subsequent cross-modal fusion.

Thirdly, we refine the Feature Pyramid Network (FPN) and Path Aggregation Network (PAN) fusion pathway and detection head by introducing residual fusion strategies. This enhancement effectively improves cross-scale feature complementarity, particularly boosting detection accuracy for both large and small targets in complex UAV scenarios.

The proposed method achieves 61.2% and 62.1% in mAP_50:95_ on the DroneVehicle and LLVIP datasets. It significantly mitigates the missed detection issue and demonstrates the effectiveness of the DAP module in intricate environments. While maintaining real-time performance, it balances accuracy and practicality, offering a viable solution for UAV dual-modality object detection.

## 2. Methods

### 2.1. Overall Architecture Design

**Figure 1:**
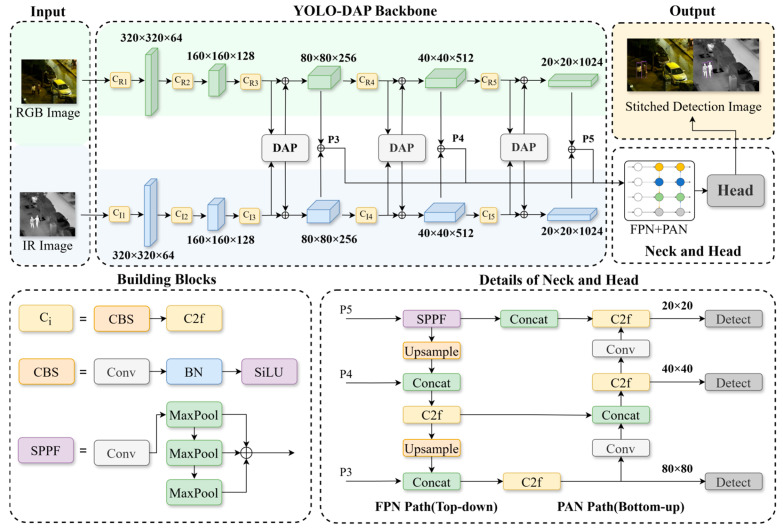
The comprehensive architecture of our proposed YOLO-DAP network. The model processes RGB and Infrared (IR) inputs through a dual-stream backbone, where the RGB stream modules are colored light green and the IR stream modules are light blue. Our proposed Dual-modality Adaptive Pyramid Transformer (DAP) modules, shown as white rectangles, are inserted at feature levels P3, P4, and P5 to perform adaptive cross-modal fusion. The fused features are then processed by a neck structure composed of a top-down Feature Pyramid Network (FPN) and a bottom-up Path Aggregation Network (PAN). In the neck and head, key components such as upsampling, concatenation, and detection blocks are colored yellow, green, and gray, respectively. The definitions of fundamental building blocks (e.g., C2f, SPPF) and their constituent colors are provided in the “Building Blocks” legend within the figure.

This work builds an end-to-end detection architecture based on the YOLOv8 framework to overcome ambiguous multi-scale target representation and inadequate modal adaptability in complex situations for UAV payload target detection [17]. Our design philosophy is guided by two core principles: first, preserving modal independence during feature extraction to maintain the integrity of modality-specific information, and second, achieving adaptive fusion through a joint attention mechanism that intelligently integrates complementary features rather than naively mixing them. The architecture is optimized through three key stages: independent feature extraction, adaptive cross-modal fusion, and multi-scale accurate detection. It is primarily composed of a dual-modality independent dual-stream backbone network, a multi-scale DAP cross-modal fusion module, and an optimized feature pyramid detection head. Figure 1 depicts the network.

In particular, the separate dual-stream backbone network extracts modality-specific characteristics from visible light and infrared pictures. This structure strictly enforces our first principle of modal independence. Due to the heterogeneity between visible light texture details and infrared thermal radiation characteristics, separate processing pathways are crucial to prevent feature contamination. Unified stride down-sampling ensures the cross-modal characteristics are spatially aligned, preparing them for fusion. To address the issue of traditional CNNs failing to capture cross-modal long-range dependencies, the DAP module is integrated into the multi-scale pyramid. This module embodies our second principle of adaptive fusion. The Transformer’s self-attention mechanism overcomes receptive field limitations, while 8 × 8 anchor pooling addresses the computational complexity, enabling a collaborative optimization of global modeling and efficiency. A critical design choice in our architecture is the strategic placement of the DAP modules exclusively at the P3, P4, and P5 feature levels, deliberately bypassing the shallower P1 and P2 layers. This decision is grounded in a crucial trade-off between semantic value and computational efficiency. From a semantic perspective, the deeper P3–P5 layers provide abstract, high-level features that are rich in contextual information (e.g., object parts and shapes), making them ideal for meaningful cross-modal fusion. In contrast, the P1 and P2 layers contain high-resolution but low-level features, such as edges and textures, where direct fusion offers limited semantic benefit and is more susceptible to noise. From a computational standpoint, the self-attention mechanism’s complexity is quadratic with respect to the number of input tokens (i.e., the spatial resolution of the feature map). Applying it to the high-resolution P1 and P2 feature maps would lead to prohibitive computational and memory costs, compromising the model’s real-time performance. Therefore, targeting P3–P5 allows our DAP module to efficiently model long-range, cross-modal dependencies at the most semantically relevant stages of the network, striking an optimal balance between performance and efficiency.

Finally, the optimized FPN + PAN feature pyramid and detection head strengthen complementary fusion of features at different scales, addressing the problem of imbalanced detection accuracy between large and small targets in UAV scenarios and forming a complete closed-loop from modality-specific feature extraction to cross-scale accurate detection.

#### 2.1.1. Dual-Modality Independent Dual-Stream Backbone Network

To strictly enforce modal independence and preserve exclusive characteristics, our architecture employs two parallel, non-sharing streams for the visible and infrared branches [18], as depicted in Figure 1. Both streams are constructed using the building blocks of the YOLOv8n architecture, selected for its computational efficiency. The detailed configuration of each stream is presented in Table 1.

Visible and infrared images are first standardized and then subjected to multi-scale down-sampling, generating five levels of feature maps (P1 to P5). The streams share an identical structure to ensure strict spatial alignment of cross-modal features, laying the foundation for subsequent fusion. Specifically:

The low-level stage, which outputs the P3 feature map (80 × 80), utilizes 3 C2f modules to strengthen spatial details like edges and textures, adapting to the contour detection of small targets.

The mid-level stage, outputting the P4 feature map (40 × 40), enhances semantic expression through 6 C2f modules to handle category discrimination of medium-scale targets.

The high-level stage, producing the P5 feature map (20 × 20), uses 3 C2f modules and subsequently introduces an SPPF module to aggregate global context, addressing the detection of large targets in complex backgrounds [19].

After this stage-wise feature extraction, the outputs (P3, P4, P5) from both streams are fed into our DAP modules for cross-modal fusion, as described in the next section.

#### 2.1.2. Multi-Scale Feature Pyramid and Detection Head Optimization

The dual-modal features enhanced by the DAP module undergo cross-scale fusion through the neck network (FPN + PAN): in the top-down path, high-level features (P5) are up-sampled and concatenated with middle-level features (P4) to enhance semantic information of small targets; the middle-level features are further up-sampled and fused with low-level features (P3) to preserve spatial details for small targets. In the bottom-up path, low-level features are down-sampled via Conv layers and concatenated with middle and high-level features to strengthen the localization accuracy of large targets. The detection head adopts the decoupled design of YOLOv8, separating classification and regression branches: the P3 branch relies on low-level details to detect small targets at the 20 × 20 pixel level, while the P5 branch utilizes high-level semantics to identify large targets at the 200 × 200 pixel level, achieving targeted detection of multi-scale targets.

### 2.2. Dual-Modality Adaptive Pyramid Transformer Module

With the development of Transformer, the Cross-Fusion Transformer (CFT) module [20] pioneered introducing self-attention into cross-modal fusion via 8 × 8 fixed anchor pooling, opening a global modeling paradigm. However, in UAV scenarios with extreme target scale differences, CFT shows obvious mismatches.

Its fixed 8 × 8 pooling erases sparse details of small targets and truncates large targets’ contextual information. Meanwhile, the unified attention strategy fails to adapt to feature pyramid layers: it mixes background noise in low-level layers and covers large targets incompletely in high-level layers, leading to poor small-target capture and insufficient large-target context.

To address these issues, this work designs the Dual-modality Adaptive Pyramid Transformer (DAP) module, which adapts to YOLOv8’s dual-stream backbone for multi-scale adaptation and cross-modal alignment.

To clarify the architectural novelty of our approach, it is crucial to contrast it with prior fusion transformers. The pioneering CFT model, for instance, applies cross-attention at a single, deep feature level. While effective for general fusion, this single-scale approach is ill-suited for UAV imagery, where target scales vary dramatically. Its fixed fusion policy struggles to preserve fine details of small targets while simultaneously capturing the global context of large ones.

Our DAP module introduces two fundamental advancements over such designs. First, unlike the single-scale nature of CFT or the more uniform multi-level processing in models like C^2^Former, DAP employs an adaptive pyramid structure. This allows it to explicitly and dynamically model cross-modal relationships at multiple scales, balancing local and global context as needed. Second, and perhaps more critically, is the distinction in feature preservation. Prior methods like CFT and C^2^Former typically replace the original modal features with the fused output. In stark contrast, our DAP module utilizes a residual enhancement strategy. The attention-fused information is added back to the original feature streams, thereby preserving their unique characteristics while injecting complementary knowledge. This dual design of adaptive multi-scale fusion and residual feature preservation forms the core architectural contribution of our work, enabling both high performance and robustness.

**Figure 2:**
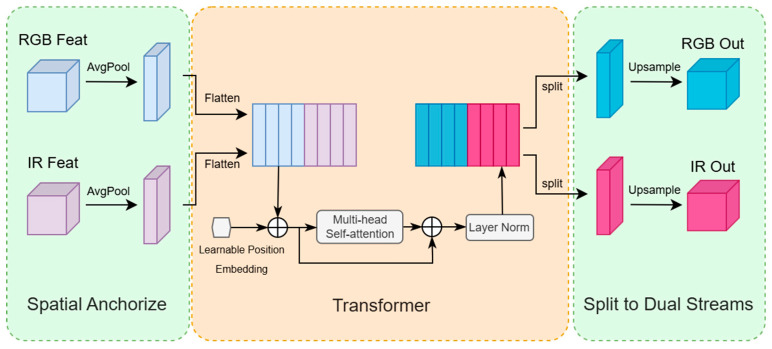
Data Flow of the Dynamic Attention Projector (DAP) Module. The DAP module enhances cross-modal features by first transforming the input RGB (light blue) and IR (light purple) feature maps into 1D vectors using Average Pooling and Flatten operations. These vectors are then concatenated, infused with Learnable Position Embedding, and processed by a Multi-head Self-attention block to dynamically capture correlations between the modalities. After passing through Layer Normalization, the resulting feature sequence is split back into its respective RGB and IR streams. Finally, each stream is Upsampled to restore its original spatial dimensions, yielding enhanced dual-modal feature maps (RGB Out, IR Out). The circle with a plus sign denotes the element-wise addition operation, which is used here to add the position embedding to the input tokens. This symbol represents the same operation in Figure 1.

While it also employs an 8 × 8 anchor pooling grid, this size was empirically chosen for its optimal balance of performance and efficiency (see Section 3.2.2), and its application within our pyramid structure overcomes the limitations of a fixed, non-adaptive approach. Its structure is shown in Figure 2. To enable the self-attention mechanism to process spatial information, we incorporate a Learnable Position Embedding. Since the standard self-attention operation is permutation-invariant and lacks inherent spatial awareness, we add a unique, learnable vector to each feature token. These embedding vectors are optimized during training, allowing the model to learn the most effective spatial representations for the cross-modal fusion task.

It is crucial to clarify the conceptual role of this module. While it computes a shared attention matrix by jointly processing features, its purpose is not to ‘contaminate’ or blindly mix modalities. Instead, it functions as an adaptive gating mechanism that learns a dynamic, spatially aware policy for fusion. It determines how much ‘trust’ to place in each modality at each location, effectively suppressing noise and leveraging complementary strengths.

#### 2.2.1. Cross-Modal Feature Encoding Processing

The DAP module takes dual-modal features of the same scale (visible light $F_{R}$ and infrared $F_{T}$) as input. First, adaptive anchor pooling is used to uniformly reduce the spatial resolution to a fixed low-dimensional size, with the calculation formula as follows:

(1) $$F_{m}^{\prime }=\mathrm{AvgPool}\left(F_{m},\mathrm{size}=\left(8,8\right)\right),$$

where m∈{R,T} denotes the modality, representing the RGB and IR streams, respectively.

This operation reduces the number of feature points from H×W to 64, thereby reducing the computational complexity of self-attention from $O\left({\left(H\times W\right)}^{2}\right)$ to $O\left(64^{2}\right)$ fundamentally avoiding computational explosion caused by high-resolution inputs. The pooled features are flattened into sequences and concatenated along the channel dimension to form a dual-modal input sequence $T\in R^{B\times 2\times 64\times C}$, where B is the batch size and C is the number of channels.

The pixel span of UAV targets ranges from 10 × 10 to 200 × 200. The 8 × 8 anchors can not only cover the local details of small targets (10 × 10 pixels) in the P3 layer but also aggregate the global context of large targets (200 × 200 pixels) through anchors in the P5 layer, balancing the feature granularity of targets at different scales. Moreover, the computing power limitation of UAV embedded platforms (single-frame inference time less than 30 ms) requires controllable feature dimensions. The 8 × 8 anchors reduce the self-attention computation from $O\left({\left(H\times W\right)}^{2}\right)$ to $O\left(64^{2}\right)$, and combined with the lightweight backbone of YOLOv8, the overall model inference speed is maintained at 18.1 ms (55.25 FPS), meeting real-time requirements.

#### 2.2.2. Multi-Head Self-Attention and Modal Interaction Mechanism

The DAP module adopts multi-head self-attention [21] to simultaneously model intra-modal long-distance dependencies and inter-modal semantic interactions, capturing the global context of single-modal features (e.g., cross-region texture correlations of vehicles in visible light, thermal signal distribution of pedestrians in infrared) and enhancing the global consistency of single-modal features [22].

Through the cross-modal sub-blocks of the attention matrix, the interaction weights between visible light and infrared features are dynamically calculated (e.g., the activation intensity of infrared heat sources in low-light regions of visible light at night), achieving cross-modal semantic alignment. For example, when visible light images are blurred due to strong reflections, the DAP module enhances the feature response of the corresponding region through infrared thermal signals, improving detection robustness.

The calculation process of attention is as follows:

(2) $$Q=TW^{Q},$$

(3) $$K=TW^{K},$$

(4) $$V=TW^{V},$$

(5) $$\mathrm{Attn}=\mathrm{Softmax}\left(\frac{QK^{T}}{\sqrt{d_{k}}}\right)V$$

where Q, K, and V are the Query, Key, and Value matrices, respectively. They are generated by projecting the input feature map T through learnable linear projection matrices $W^{Q},W^{K},W^{V}\in R^{d\times d_{k}}$.

$QK^{T}$ computes the dot-product similarity score between each query and all keys.

$d_{k}$ is the dimension of the key vectors. The scaling factor $\frac{1}{\sqrt{d_{k}}}$ is applied to prevent the dot-products from becoming too large, which helps stabilize gradients during training.

The Softmax function is applied to the scaled scores to produce the attention weights, which represent the importance of each value vector.

Attn is the final output, computed as the weighted sum of the Value vectors based on the attention weights.

By employing a multi-head mechanism with 8 parallel attention heads, the model can jointly attend to information from different representation subspaces, effectively capturing complex cross-modal correlations.

#### 2.2.3. Residual Fusion and Preservation of Modal Independence

GFD-SSD realizes dynamic modal weighting for multispectral pedestrian detection through a dual-SSD architecture and a gated fusion unit. While it outperforms two-stage methods in real-time performance, it has limitations in terms of adaptability to UAV multi-scale targets and robustness to modal registration [23].

To prevent cross-modal fusion from disrupting the original distribution of single-modal features, the DAP module adopts a residual connection strategy: the attention output is restored to the original scale via bilinear interpolation and then added back to the original modal branch in a residual manner:

(6) $$F_{m}^{\mathrm{out}}=F_{m}+\mathrm{Upsample}\left(\mathrm{Attn}\right),$$

where m∈{R,T} denotes the modality, representing the RGB and IR streams, respectively.

This design preserves the independence of visible light texture details and infrared thermal signal features, while simultaneously injecting cross-modal complementary information (e.g., infrared contours enhance the edge detection response of visible light), thus forming a dual representation of “modal-specific features + cross-modal enhancement”.

## 3. Experiments

### 3.1. Experimental Setup

#### 3.1.1. Experimental Parameters

The experiments were conducted on a workstation equipped with an AMD Ryzen 7 9700X 8-Core Processor (Advanced Micro Devices, Inc., Santa Clara, CA, USA), 32.0 GB of RAM, and an NVIDIA GeForce RTX 4060 Ti GPU (NVIDIA Corp., Santa Clara, CA, USA). The software environment consisted of Python 3.8 (Python Software Foundation, Wilmington, DE, USA), PyTorch 2.0 (Meta AI, Menlo Park, CA, USA), CUDA 11.6, and cuDNN 8.6. The model implementation was based on the Ultralytics YOLOv8 (version 8.3.156; Ultralytics, Los Angeles, CA, USA) open-source library. For data loading, the number of worker processes was set to 4 to ensure efficient training.

The basic model is YOLOv8n, and the self-developed DAP module creates a dual-modality detection architecture. Hierarchical attention fusion and multi-scale improvement of cross-modal features are enabled by embedding DAP modules at the dual-stream backbone network’s P3/P4/P5 feature scales.

Designing training hyperparameters and data augmentation schemes involves three main factors.

First, we use SGD optimiser and warm-up scheduling for the optimiser and training schedule. Momentum rises from 0.8 to 0.937 in the first three epochs, which warm up. These settings maintain convergence throughout training.

Second, we create a data augmentation process to balance model robustness and modality features. The algorithm involves 50% horizontal flipping, ±0.015 hue perturbation, ±70% saturation scaling, and ±40% brightness scaling.

Third, we disable mixup augmentation to prevent cross-modal interference. This prevents feature interference between cross-modal samples and safeguards cross-modal data.

#### 3.1.2. Datasets

Two public datasets were used to verify the algorithm’s performance: DroneVehicle [24], a UAV-view cross-modal vehicle detection dataset, and LLVIP [25], a low-light RGB-infrared pedestrian detection dataset. Both provide high-quality cross-modal image pairs with refined annotations, covering complex illumination and scene conditions, making them suitable for evaluating the robustness of dual-modality target detection algorithms.

DroneVehicle, the first large-scale dataset for UAV-view RGB-infrared cross-modal vehicle detection, was constructed by a team from Tianjin University. There are 28,439 pairs of aligned RGB-infrared photos (56,878 total) with 953,087 annotated orientated bounding boxes for 5 vehicle categories. The datasets included daytime, nighttime, and extremely dark nighttime illumination from urban roads, residential areas, parking lots, and highways. UAV flight altitude extends from 80 to 120 m, with varying viewing angles (0°, 15°, 30°, 45°) and diverse target pixel sizes (10 × 10 to 200 × 200 pixels).

LLVIP is an RGB-infrared paired dataset designed specifically for low-light vision tasks, released by a team from Beijing University of Posts and Telecommunications. The 16,836 pairs of rigorously spatio-temporally matched photos (33,672) focus on low-light conditions including nighttime streets and unlit places. Images were recorded continuously using a stereo camera and carefully registered for consistent field of view and size. Images fusion and low-light pedestrian detection are possible using the datasets’ pedestrian target annotations.

DroneVehicle tests the algorithm’s capacity to detect multi-scale vehicles from UAV images using oriented annotations and complex illumination situations to assess cross-modal features’ robustness to target orientation and low visibility. LLVIP tests pedestrian recognition in low-light conditions using rigorously aligned cross-modal images and reverse annotation methods to evaluate dual-modality fusion effects. They cover common UAV and ground monitoring cross-modal detection scenarios to test the algorithm’s generalization.

#### 3.1.3. Evaluation Metrics

In target detection tasks, scientific and comprehensive evaluation of model performance is crucial. This work adopts a general target detection evaluation system in the field of computer vision, which starts from two key dimensions—localization accuracy and classification performance—and can accurately measure the actual performance of the model. The following details several core evaluation metrics in this system.

Precision (P) and Recall (R) are fundamental and important metrics for measuring the performance of target detection models, reflecting the model’s capabilities from different perspectives.

Precision mainly reflects the model’s ability to resist false detection, i.e., the proportion of true positives among all detected positives. Recall measures the model’s ability to capture real targets, i.e., the proportion of true positives correctly detected by the model among all actual positives. The definitions of precision and recall are as follows:

(7) $$P=\frac{\mathrm{TP}}{\mathrm{TP}+\mathrm{FP}}$$

(8) $$R=\frac{\mathrm{TP}}{\mathrm{TP}+\mathrm{FN}}$$

where TP denotes the number of target instances correctly detected by the model (i.e., instances that the model identifies as positive and are actually positive); FP represents the number of incorrectly detected target instances (i.e., instances that the model misclassifies as positive but are actually negative); FN refers to the number of missed target instances (i.e., instances that are actually positive but not detected by the model).

Average Precision (AP) is a metric that comprehensively considers the model’s performance across different categories, further refined into single-threshold mAP (mAP_50_) and multi-threshold mAP (mAP_50:95_) to meet different evaluation needs.

Single-threshold mAP (mAP_50_) is mainly used to evaluate the model’s performance under loose localization conditions. It measures the model’s performance by calculating the average precision at an Intersection over Union (IoU) threshold of 0.5. IoU is the ratio of the area of intersection to the area of union between the predicted bounding box and the ground-truth bounding box; a prediction is considered correct when IoU ≥ 0.5. Its calculation formula is:

(9) $${mAP}_{50}=\frac{1}{C}\sum_{c=1}^{C}{\mathrm{AP}}_{c}\left(\mathrm{IoU}\;=\;0.5\right)$$

where C is the number of target categories, and ${\mathrm{AP}}_{c}$ is the average precision of the c-th category, obtained by integrating the precision-recall curve.

Multi-threshold mAP (mAP_50:95_) more strictly evaluates the model’s comprehensive performance under different localization accuracy requirements, especially suitable for scenarios such as UAV detection that demand high precision in target bounding boxes. It calculates the average precision across 10 IoU thresholds from 0.5 to 0.95 (with a step of 0.05). Its calculation formula is:

(10) $${mAP}_{50:95}=\frac{1}{10}\sum_{t=0}^{9}\mathrm{AP}\left(\mathrm{IoU}=0.5+0.05t\right)$$

This means that the average precision at IoU thresholds of 0.5, 0.55, 0.6, …, 0.95 must be calculated separately, and then the average of these 10 values is taken. This method can comprehensively examine the model’s performance under different localization strictness. For example, when the IoU threshold is high, the model needs to localize targets more precisely, and the average precision at this time can reflect the model’s ability in high-precision localization [26].

In summary, precision, recall, single-threshold mAP, and multi-threshold mAP complement each other, forming a relatively complete evaluation system for target detection models, which helps comprehensively and accurately assess model performance.

### 3.2. Ablation Experiments

#### 3.2.1. Ablation Study on Model Components

Ablation experiments on the DroneVehicle dataset were conducted to validate our design, specifically to assess how the model balances adaptive fusion with modal independence. Table 2 presents the results, dissecting the contribution of each component.

We established two baselines for comparison. A single-modality model using only IR dataset has a performance floor with a mAP_50:95_ of 53.7%. A second baseline with naive feature concatenation improved this score to 57.1%, confirming the general benefit of dual-modality input but highlighting the need for a more sophisticated fusion method.

We then isolated the effects of our two main contributions: the Hierarchical Self-Attention (HSA) mechanism and the Residual Fusion (RF) strategy. First, we integrated the HSA module without the residual connection. In this “hard fusion” configuration, the fused features directly replace the original ones. This setup yielded a mAP_50:95_ of 61.4%, demonstrating the attention mechanism’s effectiveness in capturing cross-modal correlations. However, such an approach risks overwriting valuable modality-specific information and can introduce noise from a less reliable modality into the fused representation.

Our complete model, integrating both HSA and RF, achieved the best overall performance with a mAP_50:95_ of 61.2%. While the mAP gain over the HSA-only model is modest, a closer look at the metrics reveals a more robust detector. The full model’s precision improved to 82.0% from the 80.6% of the HSA-only model, while maintaining a high recall of 81.6%. This indicates that the residual connection stabilizes the fusion process, preventing over-aggressive updates that could lead to false positives.

These findings empirically support our two-stage design. The HSA-only experiment confirms the benefit of joint attention, while the full model’s balanced performance shows that residual fusion is essential for preserving modal integrity. The RF strategy allows the original features to form the main information pathway, with the attention module providing a supplementary, learned enhancement. In this way, the model learns an optimal fusion policy without discarding the original, uncontaminated features. This combination proves effective for robust dual-modality object detection in UAV imagery.

**Figure 3:**
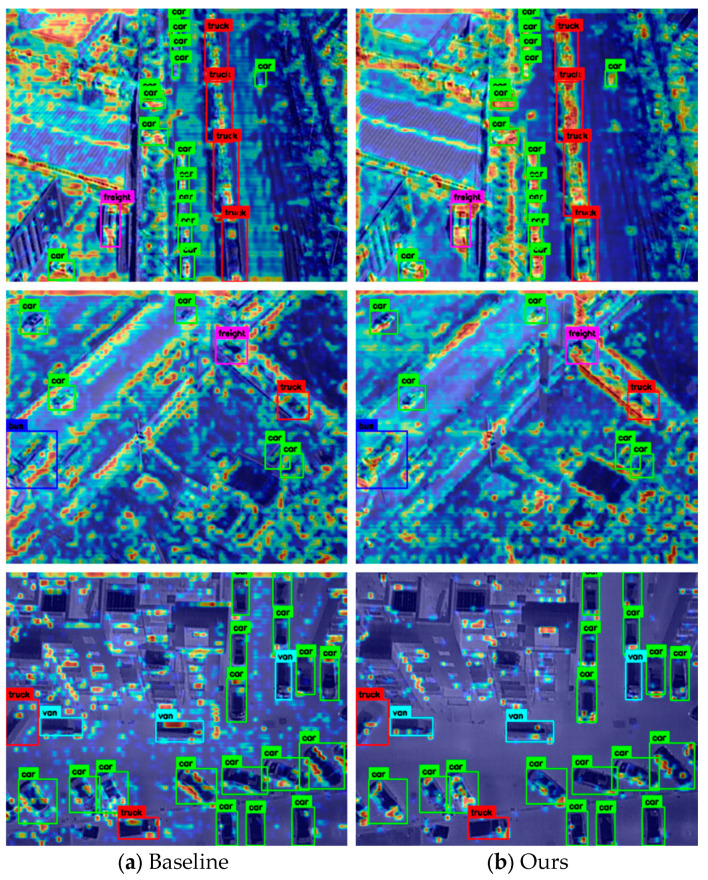
Comparative heatmap visualization. In these heatmaps, warmer colors (e.g., red and yellow) represent higher model activation, while cooler colors (e.g., blue) indicate lower activation. (**a**) The baseline model shows diffuse activation, with feature responses bleeding into the background. (**b**) Our DAP model demonstrates significantly more concentrated activation on the target objects. This visual improvement is quantified by our proposed Target Concentration Score (TCS). Our model achieves 14.32%, representing a 58.1% relative improvement over the baseline’s 9.06%, confirming a superior feature-focusing capability.

In Table 2, ↑ indicates that higher values are better, while ↓ indicates that lower values are better. √ and × denote whether the corresponding module is used or not, respectively. Figure 3 presents comparative heatmap visualizations of the DAP model (Ours) and the baseline model on the DroneVehicle dataset. Heatmaps, where color intensity indicates feature response strength (warmer colors for higher target-related activation), reveal a clear distinction. The baseline model exhibits diffused activation, leading to blurred target boundaries and interference from background clutter. In contrast, our DAP model achieves highly concentrated, high-intensity activation squarely on the targets.

To quantitatively validate this visual improvement, we introduce the Target Concentration Score (TCS), defined as the normalized average activation intensity within the ground-truth bounding boxes. Our analysis reveals that the DAP model achieves a TCS of 14.32%, a substantial 58.1% relative improvement over the baseline’s score of 9.06%.

It is important to contextualize these absolute scores. The feature maps visualized here are intermediate representations from the fusion neck, designed to provide strong discriminative cues for the final detection heads, rather than to act as pixel-perfect segmentation masks. Therefore, the key insight lies not in the absolute values themselves, but in the significant relative gain. The fact that our model boosts target concentration by over 58% at this critical fusion stage provides robust evidence of its superior feature-focusing capability.

This powerful combination of qualitative and quantitative evidence verifies DAP’s efficacy in strengthening cross-modal feature representation for UAV target detection.

#### 3.2.2. Ablation Study on Anchor Pooling Size

To validate our choice of an 8 × 8 grid for anchor pooling, we conducted a targeted ablation study to analyze the impact of different grid sizes on both model performance and inference speed. As this component does not alter the number of trainable weights, the model parameters remained constant at 28.51 M across all configurations. We tested three settings: a coarser 4 × 4 grid, our proposed 8 × 8 grid, and a finer 16 × 16 grid. The detailed results, including performance metrics and computational costs, are presented in Table 3.

The experimental results in Table 3 unequivocally demonstrate that the 8 × 8 grid offers the best trade-off.

Specifically, the 8 × 8 grid achieves the highest accuracy on both mAP_50_ (85.0%) and mAP_50:95_ (61.2%) metrics.

Intriguingly, despite the 4 × 4 grid having lower theoretical computation (GFLOPs), our 8 × 8 grid achieves a significantly higher real-world inference speed, boosting FPS from 53.48 to 55.25. This suggests the 8 × 8 configuration allows for more efficient hardware utilization.

Conversely, the 16 × 16 grid not only fails to improve accuracy but also incurs a substantial efficiency penalty. Its computational load (GFLOPs) increases by 37%.

In conclusion, the 8 × 8 anchor pooling grid is the superior choice, as it simultaneously delivers the highest detection accuracy and the fastest inference speed in our experiments. This empirical evidence provides a strong justification for our architectural design.

### 3.3. Comparative Experiments

To verify the effectiveness of the DAP method, we tested and compared it with single-modality detection methods and advanced multispectral object detection methods in recent years on DroneVehicle datasets and LLVIP datasets.

To ensure the reliability and robustness of our results, our model was conducted 3 times with different random seeds. The reported metrics in our tables are the mean and standard deviation of these runs.

#### 3.3.1. DroneVehicle Datasets

To provide both a quantitative and an intuitive comparison of our model against existing methods, we first present the absolute performance in Table 4. These raw numbers offer a detailed, concrete foundation for evaluation.

**Figure 4:**
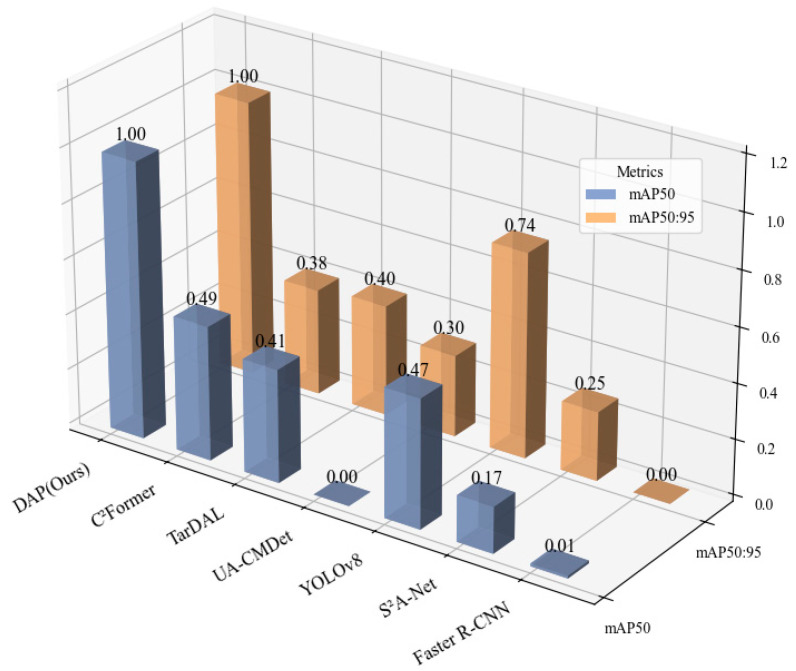
Comparative detection performance on the DroneVehicle dataset. This 3D bar chart compares our proposed DAP model with several other methods. The *X*-axis lists the models under evaluation, while the *Y*-axis indicates the achieved performance score. The two metrics, distinguished along the *Z*-axis, are mAP_50_ (blue bars), representing mean Average Precision at an IoU threshold of 0.50, and mAP_50:95_ (orange bars), the average mAP across IoU thresholds from 0.50 to 0.95. The results highlight the superiority of our DAP model, as it achieves the highest scores in both metrics, demonstrating its enhanced accuracy and localization precision. The bars are rendered with semi-transparency for better visibility in the 3D space, which may cause their apparent color to differ slightly from the solid colors shown in the legend.

To further intuitively compare the performance disparities between existing models and our proposed model, three representative models were selected, respectively, from the single-modal and dual-modal categories. All detection metrics of these selected models, along with those of our model, were subjected to normalization processing to eliminate the influence of dimension differences among metrics. Subsequently, the normalized results were visualized as Figure 4, which enables a clear and straightforward illustration of the relative performance advantages of our proposed model against the selected baseline models.

It can be seen that, for single-modality detection methods, the performance of detection using infrared images as input is significantly higher than that using visible light images. This is because the datasets contains certain low-light and dim conditions, where texture features and details in visible light images are severely degraded, making visible light-based detectors (such as YOLOv8) unable to effectively extract target edge information. In contrast, through dual-input of infrared and visible light images with fusion in the network—for example, the fusion method UA-CMDet [24] only achieves a 9% higher mAP_50:95_ than the Faster R-CNN network with only infrared input. With the continuous improvement of dual-modality networks, detection accuracy has been increasing. However, the YOLOv8 model under infrared modality still outperforms AR-CNN [30] and TarDaL [31] methods by 2.2% and 1.2% in mAP_50_, respectively. The C^2^Former method only outperforms the YOLOv8 model by 0.4% in mAP_50_. Based on the YOLOv8 backbone, our model introduces a dual-modality adaptive Transformer module, and through hierarchical cross-modal attention and residual fusion, achieves 85.0% and 61.2% in mAP_50_ and mAP_50:95_, respectively. This represents an improvement of 10.8% and 18.4% compared to the C^2^Former method, and 11.2% and 7.5% compared to the infrared-modality YOLOv8. The improvement of DAP in mAP_50_ mainly stems from the enhanced local attention of the hierarchical attention mechanism module to small targets in the P3 layer, which focuses on the edge features of 10 × 10 pixel-level vehicles through 8 × 8 anchor pooling. In contrast, C^2^Former adopts a unified attention strategy, causing small target details to be overwhelmed by the context of large targets. Meanwhile, residual fusion preserves the integrity of infrared thermal signals (such as the thermal contour of vehicles at night) through the dual representation of original features and cross-modal enhancement, avoiding the loss of modal information caused by direct feature replacement in C^2^Former. This indicates that our model effectively suppresses infrared noise and enhances the feature saliency of low-light regions in visible light images.

#### 3.3.2. LLVIP Datasets

Table 5 presents the performance of various models on the LLVIP datasets (low-light pedestrian detection). Characterized by strictly aligned RGB-infrared image pairs, 80% of the samples in this dataset were captured in extremely dark environments. Visible light images often lose pedestrian textures due to underexposure, while infrared images provide clear thermal contours, making it an ideal scenario to verify the effectiveness of cross-modal fusion for low-light target detection.

**Figure 5:**
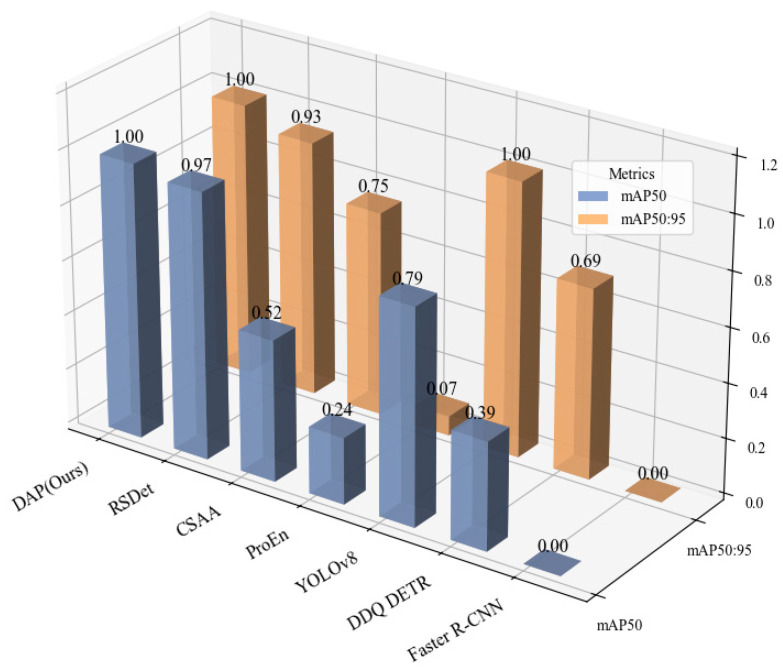
Comparative detection performance on the LLVIP dataset. This 3D bar chart compares our proposed DAP model with several other methods. The *X*-axis lists the models under evaluation, while the *Y*-axis indicates the achieved performance score. The two metrics, distinguished along the *Z*-axis, are mAP_50_ (blue bars), representing mean Average Precision at an IoU threshold of 0.50, and mAP_50:95_ (orange bars), the average mAP across IoU thresholds from 0.50 to 0.95. The results highlight the superiority of our DAP model, as it achieves the highest scores in both metrics, demonstrating its enhanced accuracy and localization precision. The bars are rendered with semi-transparency for better visibility in the 3D space, which may cause their apparent color to differ slightly from the solid colors shown in the legend.

To align with the analysis logic of LLVIP datasets, we also selected three models each from single-modal and dual-modal categories, normalized their detection metrics together with our model, and visualized the results as Figure 5 to clearly show our model’s performance advantages.

In the LLVIP datasets, approximately 80% of the scenes were captured in low-light environments, where the detection performance using infrared images as input remains superior to that using visible light images. Early fusion methods such as GAFF [34] achieve RGB + IR input through feature concatenation, with mAP_50_ reaching 94%, showing a significant improvement in accuracy compared to single-infrared modality models. However, they still exhibit obvious performance deficiencies compared to advanced single-modality detectors like YOLOv5 and YOLOv8, indicating that simple feature concatenation cannot fully exploit cross-modal complementarity.

With the deepening of research on multi-modal fusion, the ProEn model attempts to enhance modal compatibility through lightweight feature alignment. Nevertheless, its mAP_50_ drops to 93.4% and mAP_50:95_ only reaches 51.5%, reflecting its insufficiency in suppressing noise in complex low-light conditions. CSAA introduces an attention mechanism for refined feature fusion, increasing mAP_50_ to 94.3% and mAP_50:95_ to 59.2%, but the depth of cross-modal feature interaction still fails to meet the requirements of extreme low-light scenarios.

The proposed dual-modality adaptive Transformer model DAP, based on the YOLOv8 backbone network, realizes efficient interaction of multi-scale features through hierarchical cross-modal attention and residual feature fusion. The model achieves 95.9% and 62.1% in mAP_50_ and mAP_50:95_, respectively, which are 0.1% and 0.8% higher than those of RSDet [37], and its mAP_50_ is 0.7% higher than that of the single-infrared YOLOv8 model. This verifies the adaptability of cross-modal fusion to extreme scenarios.

#### 3.3.3. Complexity Analysis on DroneVehicle

A comprehensive evaluation of object detection models for UAVs must extend beyond accuracy metrics to include practical considerations of computational cost. For real-world deployment on resource-constrained aerial platforms, model efficiency is not merely an advantage but a fundamental requirement. We therefore conducted a rigorous analysis of our DAP model’s complexity against that of prominent baselines, including recent transformer-based fusion methods such as CFT and C^2^Former.

The analysis, summarized in Table 6, employs two fundamental, hardware-agnostic metrics: the number of trainable parameters, which reflects model size, and Giga Floating-point Operations (GFLOPs), which quantifies the total computational workload for a single inference pass. GFLOPs serve as a robust indicator of the potential inference latency.

The results presented in Table 6 reveal a compelling narrative. Our DAP model not only establishes a new state-of-the-art in detection accuracy on the DroneVehicle dataset, achieving an mAP_50_ of 85.0% and an mAP_50:95_ of 62.1%, but it does so with unparalleled efficiency. It surpasses the strong CFT baseline by a significant margin of +5.9% in mAP_50_, a testament to the superior feature fusion capability of our adaptive pyramid architecture.

Perhaps more importantly for practical applications, this leap in accuracy is accompanied by a radical reduction in computational demand. Compared to CFT, our model requires a mere 12% of the computational workload and 14% of the parameters. This dramatic, nearly order-of-magnitude reduction in GFLOPs directly implies a substantial advantage in inference speed and a significantly lower energy footprint.

In conclusion, the analysis demonstrates that our DAP model fundamentally breaks the conventional trade-off between accuracy and efficiency. It simultaneously delivers state-of-the-art performance while maintaining a computational profile that is orders of magnitude leaner than competing high-performance fusion transformers. This synergy of high accuracy and operational efficiency makes DAP a uniquely powerful and deployable solution for real-world UAV object detection tasks.

#### 3.3.4. Analysis of Experimental Results

After conducting comparative experiments on the two datasets, we performed a more detailed analysis of the model data from three aspects: index change curves, PR curves, and comparisons of actual detection images.

**Figure 6:**
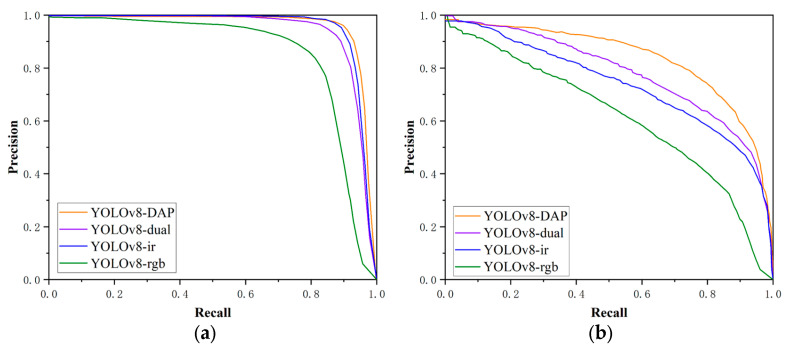
PR curves of various detection models on different datasets. (**a**) LLVIP. (**b**) DroneVehicle.

Figure 6 shows the Precision-Recall (PR) curves and trends of key indicators of various models on the DroneVehicle and LLVIP datasets. It can be seen that the PR curve of DAP is significantly superior to those of single-modality baselines (Yolov8-rgb/Yolov8-ir) and the dual-channel concatenation model (Yolov8-dual), indicating that cross-modal semantic alignment can effectively reduce the missed detection rate and false alarm rate.

Subsequently, we performed testing and validation for the multi-modal object detection task on the LLVIP and DroneVehicle datasets sequentially. Initially, we evaluated the LLVIP datasets, which concentrate on RGB-infrared dual-modality pedestrian identification in low-light conditions. It encompasses intricate working settings characterized by day-night transitions and significant illumination variations (including unlit roadways at night and dim dawn scenarios), establishing a stringent baseline for the model’s cross-modal feature adaptability and resilience.

**Figure 7:**
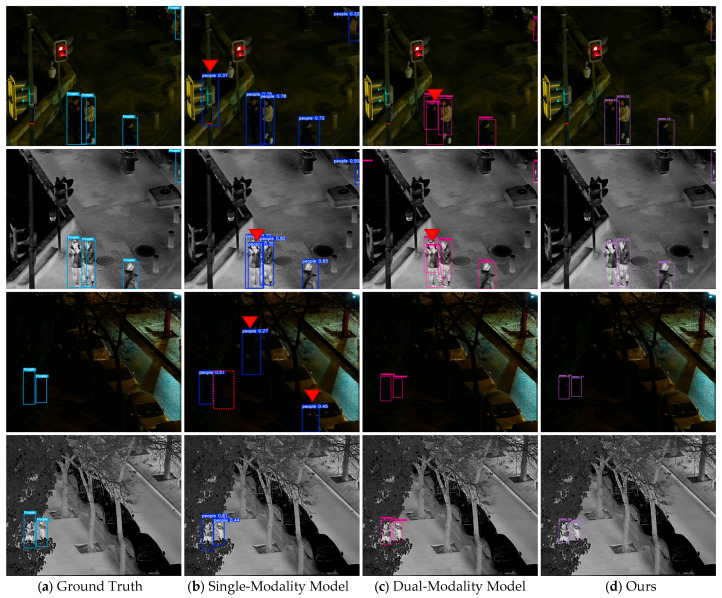
Comparison images of various detection models on the LLVIP datasets. It shows the results of different detection models on the LLVIP datasets: (**a**) Ground Truth; (**b**) detection results of single-modality models; (**c**) detection results of dual-modality models; (**d**) detection results of our method integrated with the DAP module (Ours). Red triangles mark false positive targets, and red boxes mark missed targets, which are used to quantify differences in detection performance among models.

**Figure 8:**
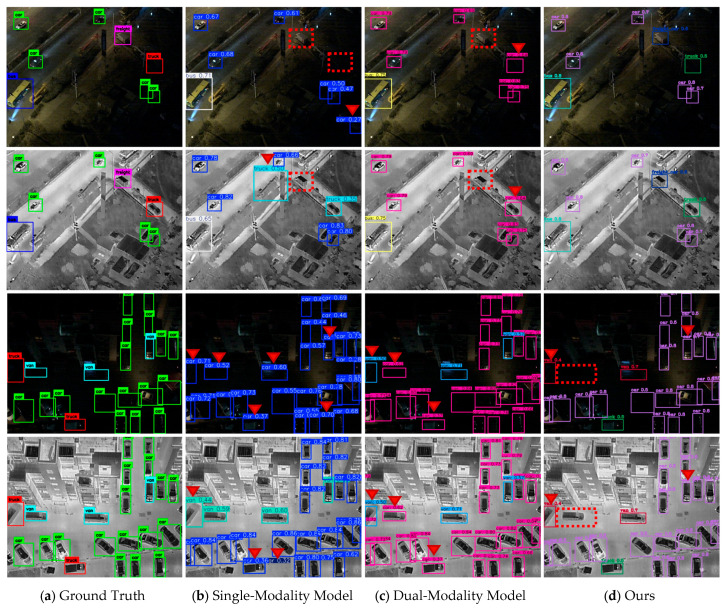
Comparison images of various detection models on the DroneVehicle datasets. It shows the results of different detection models on the DroneVehicle datasets: (**a**) Ground Truth; (**b**) detection results of single-modality models; (**c**) detection results of dual-modality models; (**d**) detection results of our method integrated with the DAP module (Ours). Red triangles mark false positive targets, and red boxes mark missed targets, which are used to quantify differences in detection performance among models.

Figure 7 and Figure 8 illustrate that single-modality models demonstrate inaccuracies and failures in pedestrian identification. For instance, utility poles in the photos are erroneously classified as pedestrians; in the visible light modality, pedestrians in poorly lit regions are inaccurately detected, while other things in the surroundings are Due to the mistakenly perceived as pedestrians. Basic dual-modality models merely execute “concatenation-style” fusion of RGB and infrared information, failing to thoroughly investigate cross-modal complimentary correlations, resulting in erroneous conclusions when confronted with identical contour interferences. Conversely, our model circumvents these problems and precisely detects all targets within the images. This illustrates that the residual fusion technique stimulates low-light areas in visible images using infrared heat sources; concurrently, it maintains infrared thermal signal characteristics and diminishes misidentifications of analogous targets via visible light texture augmentation.

Thereafter, we performed examinations on the DroneVehicle datasets. As illustrated in Figure 5, in low-light and dark conditions, visible light images experience a loss of target texture and a significant rise in background noise due to inadequate illumination, complicating the model’s ability to extract effective discriminative features. In the second photograph, all targets were erroneously identified as automobiles. Despite their ability to detect heat sources, infrared models are prone to false positives and missed detection due to inadequate resolution of heat sources. The implementation of the dual-channel model mitigated this phenomenon; however, inadequate investigation of cross-modal correlations resulted in significant instances of missed and incorrect detection. Conversely, following the implementation of our model, there were no missed or false detection in the first image, and only one missed detection and one erroneous detection in the second image. The suggested method markedly improves the robustness of target identification in low-light conditions via a cross-modal feature fusion process, exhibiting superior generalization capabilities in complicated tasks involving multi-source heterogeneous data.

The experimental findings demonstrate that DAP, utilizing hierarchical cross-modal attention and residual feature fusion, markedly surpasses conventional methods in multi-scale target detection and robustness in low-light conditions, offering an effective solution for UAV cross-modal detection. Future research may investigate dynamic attention methods to accommodate extreme conditions, enhancing the algorithm’s generalization capability in practical UAV applications.

## 4. Conclusions

This work proposes integrating the Dual-modality Adaptive Pyramid Transformer (DAP) module into the YOLOv8 framework to develop a cross-modal target detection system for unmanned aerial vehicles. The system is aimed at addressing the issues caused by multi-scale targets and complex illumination in UAV scenarios, specifically including the problems of single-modality missed detection and feature aliasing that exist in traditional fusion methods. Its core innovations are as follows: a RGB-infrared dual-stream backbone network based on CSPDarknet, where the visible light part is used to enhance edge textures and the infrared part is used to integrate thermal signal context; the DAP module embedded into the P3-P5 layers of the feature pyramid, which improves efficiency through 8 × 8 anchor pooling and combines hierarchical attention with residual fusion; and an improved FPN + PAN cross-scale fusion structure and decoupled detection head. Experimental results show that on the DroneVehicle datasets, the system achieves an mAP_50:95_ of 61.2%. On the LLVIP low-light datasets, it achieves an mAP_50:95_ of 62.1%. This study provides technical support for UAV cross-modal target detection. Future work will extend the DAP module to tasks such as industrial defect identification and rural scene target monitoring, quantify the effects of the design through ablation experiments, and optimize hyperparameters to balance the detection accuracy and real-time performance of UAV edge devices.

## Figures and Tables

**Table 1:** Architecture of a single stream in the backbone (based on YOLOv8n). Both RGB and IR streams share this identical structure before fusion. The n column denotes the number of bottleneck blocks in the C2f module.

Stage	n	Output Channels	Output Resolution	Feature Level
Input	-	3	320 × 320	-
C_i1_	-	64	160 × 160	-
C_i2_	3	128	80 × 80	-
C_i3_	6	256	40 × 40	P3
C_i4_	6	512	20 × 20	P4
C_i5_	3	1024	10 × 10	P5

**Table 2:** Ablation Study on DroneVehicle Datasets.

Configuration	Channel	HSA	RF	P	R	mAP_50_	mAP_50:95_
Yolov8 (IR-Channel)	single	×	×	70.0	71.8	73.8	53.7
Yolov8 (dual-Channel)	dual	×	×	75.4	73.7	78.2	57.1
Yolov8 (only HSA)	dual	√	×	80.6	81.9	84.7	61.4
Yolov8 (only RF)	dual	×	√	82.0	79.5	84.1	60.6
Yolov8 (HSA + RF)	dual	√	√	82.0 (↑ 12.0)	81.6 (↑ 7.9)	85.0 (↑ 6.8)	61.2 (↑ 4.1)

**Table 3:** Ablation study on the anchor pooling grid size. We compare performance (mAP) and efficiency (GFLOPs, FPS). The best configuration is highlighted in bold.

Pooling Size	mAP_50_ (%)	mAP_50:95_ (%)	GFLOPs	FPS
4 × 4	84.5	60.9	24.89	53.76
**8 × 8 (Ours)**	**85.0**	**61.2**	**27.41**	**55.25**
16 × 16	84.9	60.8	37.49	53.48

**Table 4:** Detection Performance Comparison of Models on DroneVehicle Datasets. Bold indicates the best performance in the table.

Model	Modality	mAP_50_ (%)	mAP_50:95_ (%)	References
mono-modality				
Faster R-CNN	RGB	55.9	28.4	[11]
S^2^A-Net	RGB	61.0	36.9	[27]
YOLOv5	RGB	62.1	-	[28]
YOLOv8	RGB	61.3	36.8	[29]
Faster R-CNN	IR	64.2	31.1	[11]
S^2^A-Net	IR	67.5	38.7	[27]
YOLOv5	IR	70.7	-	[28]
YOLOv8	IR	73.8	53.7	[29]
multi-modality				
UA-CMDet	RGB + IR	64.0	40.1	[24]
AR-CNN	RGB + IR	71.6	-	[30]
TarDAL	RGB + IR	72.6	43.3	[31]
C^2^Former	RGB + IR	74.2	42.8	[32]
DAP (Ours)	RGB + IR	**85.0 ± 0.1**	**61.2 ± 0.3**	This work

**Table 5:** Detection Performance Comparison of Models on LLVIP Datasets. Bold indicates the best performance in the table.

Model	Modality	mAP_50_ (%)	mAP_50:95_ (%)	References
mono-modality				
Faster R-CNN	RGB	88.8	47.5	[11]
DDQ DETR	RGB	88.3	47.0	[33]
YOLOv5	RGB	90.8	50.0	[28]
YOLOv8	RGB	91.9	54.0	[29]
Faster R-CNN	IR	92.6	50.7	[11]
DDQ DETR	IR	93.9	58.6	[33]
YOLOv5	IR	94.6	61.9	[28]
YOLOv8	IR	95.2	62.1	[29]
multi-modality				
GAFF	RGB + IR	94.0	55.8	[34]
ProEn	RGB + IR	93.4	51.5	[35]
CSAA	RGB + IR	94.3	59.2	[36]
RSDet	RGB + IR	95.8	61.3	[37]
DAP (Ours)	RGB + IR	**95.9 ± 0.1**	**62.1 ± 0.1**	This work

**Table 6:** Comparison of performance and complexity on the DroneVehicle dataset. Our model establishes a new state-of-the-art in both accuracy and efficiency. Bold indicates the best performance in the table.

Model	mAP_50_ (%)	mAP_50:95_ (%)	GFLOPs	Parameters
YOLOv8-IR	73.8	53.7	8.75	3.15 M
YOLOv8-dual	78.2	57.1	11.4	4.28 M
C^2^Former	74.2	42.8	100.9	132.5 M
CFT	79.1	59.4	224.4	206.3 M
**DAP (Ours)**	**85.0 ± 0.1**	**61.2 ± 0.3**	**27.41**	**28.51 M**

## Data Availability

Data are contained within this article.
